# Primary Shelf-Life Assessment of Fresh Vegan Spinach Potato-Based Pasta (Gnocchi) Using an Accelerated Test Approach

**DOI:** 10.3390/foods15061012

**Published:** 2026-03-12

**Authors:** Stefano Zardetto, Carlos Gabriel Arp, Gabriella Pasini

**Affiliations:** 1 Department of Agronomy, Food, Natural Resources, Animals and Environment, University of Padova, Viale dell’Università 16, 35122 Padova, Italy; carlosgabriel.arp@unipd.it (C.G.A.); gabriella.pasini@unipd.it (G.P.); 2 Comisión de Investigaciones Científicas de la Provincia de Buenos Aires, Centro de Investigación y Desarrollo en Ciencia y Tecnología de Alimentos (CIDCA), Consejo Nacional de Investigaciones Científicas y Técnicas, Facultad de Ciencias Exactas, Universidad Nacional de La Plata, 47 y 116, La Plata 1900, Argentina

**Keywords:** gnocchi, shelf-life, food quality, modified atmosphere packaging, physicochemical properties, microbiological properties

## Abstract

The primary shelf life (PSL) of fresh vegan spinach gnocchi packaged under a modified atmosphere (MAP) was investigated. Microbiological, physicochemical, and sensory properties were monitored during storage at three temperatures (4, 8, and 12 °C). The microbial load remained below the limit considered safe (3 log CFU g^−1^) in all samples during storage at all tested temperatures. Storage time significantly increased the hardness of uncooked gnocchi (*p* < 0.05) and the water absorption index (*p* < 0.05). Moreover, at higher storage temperatures, the kinetic rate of hardness decreased in uncooked gnocchi (0.29 N day^−1^ at 12 °C vs. 0.35 N day^−1^ at 4 °C). Conversely, in cooked gnocchi, as the storage temperature increased, the rate of hardness acceleration increased. The sensory analysis results varied according to storage temperature, and the Overall Quality Index (OQI), combined with principal component analysis (PCA), was used to determine PSL values. The Arrhenius relationship successfully described the temperature dependence of reaction rate constants, and the calculated Q_10_ value (3.0) confirmed hardness as the quality attribute most affected by temperature. OQI showed a strong correlation with cooked-gnocchi hardness, and a sensory cutoff of 6.5 was established and confirmed by the sensory panel. The corresponding hardness rejection value was 12.1 N. The PSL was estimated based on sensory and texture criteria, as microbial quality was not a limiting factor. Under non-isothermal cold-chain conditions, PSL was predicted using the time–temperature tolerance (TTT) approach, yielding a value of 42 ± 3 days.

## 1. Introduction

“Gnocchi” are an Italian fresh pasta traditionally prepared with wheat flour or semolina and mashed potatoes in home cooking. Nowadays, industrial production predominantly relies on reconstituted potato flour or flakes for gnocchi manufacture. The industrial product can be made with fresh or flaked potatoes, adding other ingredients such as soft wheat flour, rice flour, starch potato, dried egg yolk or pasteurized liquid eggs, salt flavouring, and additives [1]. Nevertheless, there is no legislation regulating their composition and production.

In recent years, to expand the gnocchi global market, alternative ingredients such as quinoa, amaranth flour, and navy bean flour have been incorporated to enhance nutritional properties. Additionally, ingredients like sweet potato, tomato, spinach, pumpkin, and broccoli have been used to improve sensory characteristics [2,3,4,5,6,7]. Therefore, gnocchi composition and processing exhibit great variability, reflected in the wide range of gnocchi types available on the market, thereby increasing consumer choice.

For this reason, the global gnocchi market has considerably increased and is expected to grow at a Compound Annual Growth Rate (CAGR) of 4.50% in the Forecast Period of 2024–2032 [8]. Moreover, the vegan trend has grown significantly in recent years, driven by concerns about health, the environment and animal welfare [9]. Spinach vegan gnocchi is a variation of traditional gnocchi, incorporating spinach for added flavour and colour without using derived animal raw materials such as egg, milk powder, whey powder, or flavouring and additives, in line with the rising trend for clean-label fresh pasta products [10].

Despite significant variability in ingredients, the industrial process for making potato gnocchi is quite standardized. Potato gnocchi are produced using a cooking machine that mixes and rehydrates the ingredients with hot water. The resulting mashed potatoes are extruded or shaped into various forms (classic shape, round shape or sunken wavy shape). These are then pasteurized, packed in thermoformed trays or flexible films, and refrigerated. Although shelf-stable gnocchi are commercially available, fresh potato gnocchi stored at 4 °C represent the primary focus of industrial production and consumer demand. To better preserve the product and extend its shelf life during storage, modified-atmosphere packaging (MAP) is widely used. This technology helps inhibit the growth of spoilage bacteria and better preserve the product’s colour [11]. Primary shelf life (PSL) is typically determined by food-spoilage reactions occurring at the different stages of product commercialization, including storage and distribution, while the package remains unopened. From a microbiological perspective, a combination of acidification and refrigerated conditions helps preserve the product. Maintaining a pH below 5.0 and a temperature below 8 °C is sufficient to prevent the outgrowth of spore-forming psychrotrophic microorganisms such as *Bacillus cereus* and non-proteolytic *Clostridium botulinum*, which can survive the heat treatment process [12]. Recently, Purgatorio et al. [13] reported that spoilage bacteria from the genus *Bacillus* are the primary contributors to spoilage of gnocchi stored at ambient temperature, particularly when acidity regulators are not used to lower the pH. Their findings identified the production environment and flour ingredients as primary sources of contamination.

Consumers value fresh gnocchi for its colour, cooking quality, and texture [6]. However, these attributes tend to deteriorate during storage, eventually reaching unacceptable levels when compared to the freshly made product. Additionally, textural characteristics may change through starch modification and product dehydration [11,14]. The colour, which is influenced by the recipe’s ingredients, can change over time [11], as can the taste, both of which further diminish consumer acceptability [2].

When a packaged food product is deemed unacceptable for consumption, the PSL is considered to have ended. This threshold can be established by monitoring the various spoilage reactions (i.e., microbiological and physicochemical) and/or their sensory parameters during storage. Consequently, the kinetics of these processes and their interrelation determine the acceptability limit [15].

A sensory panel is typically employed to estimate the PSL of a food product. For this purpose, an “indicator parameter”—previously identified as the attribute that best reflects product acceptability or unacceptability—is evaluated by trained judges. Shelf life is then determined by analysing the mean panel score as a function of storage time using the cutoff (COP) methodology. This approach allows assessment of the overall quality of a product until the PSL reaches its endpoint [16].

Based on currently available information, no studies have examined the PSL of fresh potato gnocchi by integrating physicochemical and microbiological parameters with sensory data. Most research on potato gnocchi focuses on microbiological aspects and shelf-stable formulations, or the evaluation of the quality and safety of new gnocchi formulations. Furthermore, there is a lack of data regarding the refrigerated storage of gnocchi at various temperatures. An accelerated test approach not only provides information on the relationship between quality changes and temperature but also helps identify optimal storage conditions to preserve refrigerated gnocchi’s quality characteristics.

In this context, the aim of this study was to assess sensory, physicochemical (colour and texture), and microbiological changes in fresh vegan spinach gnocchi stored under different refrigeration conditions. The study combined temperature-dependent kinetic models with sensory analysis to determine the product’s acceptability threshold and to estimate the PSL of fresh gnocchi under non-isothermal cold-chain conditions.

## 2. Materials and Methods

### 2.1. Sample Preparation

Fresh vegan spinach potato gnocchi were produced at an industrial plant (Olmo di Martellago, Venice, Italy). The formulation used in this study was provided by the manufacturer (Voltan S.p.A., Olmo di Martellago, Italy) and corresponds to the standard industrial recipe based on potato purée [17], with the addition of spinach. The main ingredients included potato flakes, soft wheat flour, 7% (*w w*^−1^) fresh spinach, 0.5% (*w w*^−1^) dehydrated spinach, salt, and lactic acid to achieve a pH lower than 5.0. The “fresh spinach” component was processed industrially through washing, cutting, blanching, salting (4% *w w*^−1^), and storage at −5 °C.

All the ingredients were kneaded in a mixer (Glass, Frankfurter, Germany) with the addition of hot water at 90 °C, followed by 15 min of mixing at a constant temperature of 80 °C. The dough was shaped using a forming machine (Facchini, MOD. GNF12, Milano, Italy). The gnocchi were then packaged in plastic bags under modified atmospheric conditions (20.0% CO_2_; <1.5% O_2_) and pasteurized at 70 °C for 20 min. After the heat treatment, the product was cooled to 5 °C within 2 h.

The packaging material (19.7 cm × 15.5 cm) was provided by SDR PACK S.P.A. (Rosà, Vicenza, Italy) and consisted of OPA (thickness 20 µm) and PP films (thickness 60 µm). Each package, containing 400 g of product, was stored for up to 67 days at 4, 8 and 12 °C. Samples from two different batches were prepared on separate days. In total, 450 samples were analysed: 300 from batch A and 150 from batch B. The samples were divided into three runs of 150 each, with 50 samples tested at each temperature. Additionally, at each sampling stage (every 7 days), samples were tested in triplicate, unless otherwise specified for a particular session.

### 2.2. Water Vapor Transmission Rate (WVTR) of the Packaging

The package’s water vapor transmission rate (WTR) was determined according to Zardetto et al. [18]. Briefly, bags with calcium chloride and a modified atmosphere were weighed and placed in a constant relative humidity chamber (SICCO^®^ Mini 1, Grünsfeld, German). A data logger was employed to monitor the temperature and relative humidity of the chamber. The chamber was kept at a stable temperature of 30.4 ± 0.2 °C. The package’s weight data over time was linearly regressed, and the WVTR value was calculated from the slope coefficient.

### 2.3. Gas Composition Measure in Headspace of Packaging

Headspace O_2_ and CO_2_ levels in the packaged fresh spinach gnocchi were determined with a PBI Dansensor O_2_/CO_2_ analyser (Checkmate 9900, Ringsted, Denmark). To prevent changes in gas composition, measurements were conducted destructively, with each package measured only once. All samples stored at the three different temperatures were analysed after 10, 17, 25, 31, 39, 46, 53, 60, and 69 days of storage.

### 2.4. Chemical Analyses of the Gnocchi

Protein, fat, and ash were determined according to the methods of ISTISAN 96/34. Total dietary fibre (TDF) content was measured according to AOAC International Method 991.43 [19] by means of the total dietary fibre kit (Megazyme, Wicklow, Ireland). Carbohydrates were calculated by difference. The chemical composition of raw gnocchi was analysed in two independent batches at time zero.

### 2.5. pH, Moisture Content, and Water Activity

The pH of raw spinach gnocchi was determined by a pH meter (mod. pH80, XS Instruments, Carpi, Italy) on homogenized samples. Moisture content was determined gravimetrically at 130 °C for 1 h according to D.M. 11/09/1967. Water activity (*a_w_*) was measured using a dew point instrument (mod. Aqualab CX3, Decagon Devices Inc., Pullman, WA, USA).

### 2.6. Microbiological Analysis

To assess PSL, the total viable count (TVC), the counts of aerobic and anaerobic spore-forming bacteria, and the pathogenic microorganisms *Salmonella* spp. and *Listeria monocytogenes* were monitored using the plate count technique. A representative gnocchi sample (10 g) was transferred to a sterile stomacher (BagMixer^®^ Interscience, Saint Nom la Bretèche, France) with 90 mL of sterilized Ringer solution (Merck, Darmstadt, Germany). Decimal progressive dilutions were prepared.

For microbiological analyses, the following conditions were applied: total viable count (TVC) on Plate Count Agar (PCA, Merck, Darmstadt, Germany) (ISO, 4833-1:2013) [20] at 30 °C for 72 h. Spore-forming bacteria (aerobic and anaerobic) were quantified according to the MFLP-44 guidelines [21]. Test tubes containing 25 mL of sample were warmed at 75 °C for 20 min, followed by plating of serial dilutions on Tryptic Soy Agar (TSA) and incubation at 35 °C for 48 h in aerobic and anaerobic conditions, depending on the species.

To detect *L. monocytogenes* and *Salmonella* spp., 25 g samples were pre-enriched in selective broth (Frazer Listeria Selective broth and LX-broth, respectively). Bacteria detection was performed using the rapid Enzyme-Linked Fluorescent Assay method (Biomérieux, Marcy l’Etoile, France) (AFNOR, BIO 12/11-03/04; AFNOR BIO 12/32-10/11).

### 2.7. Cooking Behaviour

A total of 100 g of gnocchi was cooked in 1000 mL of tap water (hardness of 40 °f, pH 7.1, total dissolved solids 280 mg L^−1^) for 3 min. Afterwards, they were drained for 5 min and weighed. The water absorption index (WAI) was calculated by using the following equation:

(1) $$WAI\;(g\;water\;100\;g^{-1})=\;\frac{weight\;of\;cooked\;pasta\;\left(g\right)-weight\;of\;uncooked\;pasta\;(g)}{weight\;of\;uncooked\;dry\;matter\;(g)}$$

Additionally, the matter loss during cooking was determined gravimetrically using the recovered cooking water, which was evaporated at 103 °C until constant weight. The residue was weighed, and the cooking loss was calculated as a percentage of the sample’s initial weight.

### 2.8. Texture Analysis

The textural characteristics of the fresh spinach gnocchi samples were determined using a texture analyser (Brookfield CT3, Middleboro, MA, USA) equipped with a 10 kg load cell and a cylindrical probe (⌀ 38  mm). Texture Profile Analysis (TPA) was performed as described by Hedayati and Mazaheri Tehrani [22], with slight modifications: pre-test speed of 2 mm s^−1^, test speed of 1 mm s^−1^, post-test speed of 5 mm s^−1^, and 40% compression at an initial detection force of 0.098 N. Hardness (N) and adhesiveness (N s) were measured. Ten replicates were performed at room temperature (21 °C) on the longitudinal side of each cooked and uncooked gnocchi sample. Samples stored at 4 °C were analysed after 10, 25, 31, 39, 46, 53, 60 and 67 days of storage; those stored at 8 °C after 10, 23, 30, 39, 47, 54, 64, and 67 days; and those stored at 12 °C after 10, 13, 23, 27, 33, 41, 47, 54, 61 and 68 days.

### 2.9. Colour Measurement

The fresh spinach gnocchi colour was determined using a Minolta CR-300 (Tokyo, Japan) colorimeter based on the CIELAB colour system. Measurements were carried out on uncooked gnocchi samples, which were minced, mixed, and placed onto the instrument’s material holder accessory. Readings were performed at three different points. The parameters a* (red/green index) and b* (yellow/blue index) were measured, and Hue angle values (*h°*) were calculated using the equation provided by Marcos et al. [23].

(2) $$h^{{}^{\circ}}={tan}^{-1}\left(\frac{b^{*}}{a^{*}}\right)$$

### 2.10. Sensory Evaluation

A panel of ten experienced and trained judges conducted the sensory evaluation of cooked vegan spinach gnocchi. The panellists were specifically selected for their expertise in the sensory analysis of fresh pasta products. In accordance with the ISO 8586:2023 [24], all judges had received appropriate training as members of the expert panel and had accumulated extensive experience in evaluating the sensory quality of fresh pasta and gnocchi (more than 700 testing sessions).

The methodology followed was based on ISO 13299:2016 [25]. Panellists assessed the following quality attributes: “green colour” (perceived intensity of the green hue typical of spinach-containing gnocchi: fresh spinach purée spread thinly on a white plate), “potato odour” (aroma associated with cooked potato: warm mashed potatoes (unsalted), freshly prepared), “spinach odour” (aroma associated with cooked spinach: blanched spinach leaves blended), “potato flavour” (taste notes characteristic of boiled potato: neutral mashed potato), “spinach flavour” (vegetal taste associated with cooked spinach: spinach purée served warm), “acidity” (taste associated with acid compounds naturally present or added for preservation: potato gnocchi acidified with lactic acid to pH 5.0), “bitterness” (taste associated with compounds producing bitterness: caffeine solutions according to ISO 3972:2011 [26]), “sweetness” (taste associated with sucrose solution according to ISO 3972:2011), “saltiness” (taste associated with sodium chloride solution according to ISO 3972:2011), “adhesiveness” (degree to which the product adheres to the palate, teeth, or tongue during chewing), and “softness” (low resistance to initial biting or compression between the molars, perceived as ease of deformation during the first chew). A five-point intensity scale was used, ranging from 1 (very slight intensity) to 5 (strong intensity) with a specific scale for the colour attribute ranging from 1 (light green) to 5 (dark green). Additionally, the Overall Quality Index of the cooked vegan spinach gnocchi was calculated from the mean intensity scores of the evaluated attributes, converted to a 0–10 scale using the following equation [27]:

(3) $$Overall\;Quality\;Index\;\left(OQI\right)=MEAN\left[Quality\;indices\right]\times 2.0\;$$

All sensory evaluations were conducted in a controlled sensory testing environment.

### 2.11. Mathematical Models Fitting for Quality Deterioration

The kinetics of quality loss were determined by analysing the experimental data as a function of time for each storage temperature.

The sensory and texture parameters were fitted by the following sigmoidal function, as proposed by Buratti et al. [28]:

(4) $$y\;(t)=a+\frac{b}{\left[1+e^{-\left(\frac{t-c}{d}\right)}\right]}$$

where *y*(*t*) is the analysed parameter, *a* is maximum shift, *b* is the transition centre, *t* is time, and *c* and *b* are constants.

The deterioration rate constant (*k*) was modelled using the Arrhenius equation [29]:

(5) $$k=k_{0}e^{-\frac{E_{a}}{RT}}$$

where *T* is the temperature in K, *E_a_* is the activation energy (J mol^−1^), and *R* is the universal gas constant (J mol^−1^ K^−1^). Values of *E_a_* were estimated from the slope of Arrhenius plots of ln (*k*) versus (1/*T*) by linear regression.

For narrow temperature ranges such as 4–12 °C, the *PSL* obtained from experimental data at each temperature can be used to calculate PSL at any other temperature using the logarithmic shelf-life model, also known as the shelf-life plot. In this model, the relationship between PSL and temperature follows a linear trend, represented by the equation below [30]:

(6) $$PSL=t_{0}e^{-bT}$$

where *t*_0_ is the *PSL* at 0 °C, *b* is the slope of the regression line and *T* is the temperature (°C).

Additionally, the reaction’s temperature sensitivity, indicated by the *Q*_10_ value, was calculated from the slope of model (*b*) using the following equation:

(7) $$Q_{10}=e^{10b}\;$$

Considering that *E_a_* indicates the minimum energy needed to trigger the reaction, the *Q*_10_ value and *E_a_* can be related by [31]:

(8) $$\ln Q_{10}=\frac{10E_{a}}{RT\left(T+10\right)\;}$$

The shelf-life plot can be used to estimate the PSL under non-isothermal cold-chain conditions, given the thermal profile to which the product may be exposed (time–temperature tolerance, TTT).

### 2.12. Statistical Analyses

One- and two-way ANOVA were performed at α = 0.05, using storage time (0–67 days) and temperature (4, 8, 12 °C) as factors. The data were statistically analysed using the Statgraphics software v. XVI.II (Statistical Graphics Corp., STSC Inc., Englewood Cliffs, NJ, USA).

## 3. Results and Discussion

### 3.1. Characterization of Raw Fresh Spinach Gnocchi

The nutritional composition and moisture content of sampled gnocchi, determined immediately after their production, are shown in Table 1.

The measured pH was 4.71 ± 0.01, and water activity (*a_w_*) at 21 °C was 0.981 ± 0.002. Since a pH lower than 5 was achieved, microbial spoilage by aerobic and anaerobic spore-forming bacteria is expected to be limited in refrigerated storage conditions (4 °C) [32,33]. The water activity value aligns with those reported in the literature for fresh potato gnocchi made from potato flakes [1].

### 3.2. WVTR of the Packaging

The WVTR value, calculated from the package weight vs. storage time linear regression slope, resulted in 0.1216 g 24 h^−1^ (*r*^2^ > 0.99). Given the packaging area of 0.061 m^2^, the WVTR value was 1.991 g water m^−2^ 24 h^−1^. The WVTR calculated for the packages was approximately four times higher than the value reported on the supplier’s technical sheet for the package material. As shown by Reinas et al. [34], there is a high contribution of the bag seals (around 25%) to the water permeability. Therefore, the packaging performance should be experimentally determined under conditions that represent the real storage conditions. Considering an average value of 60% RH in domestic refrigerators [35] and RH in the packaging headspace equal to the water activity of the gnocchi, the difference in the partial pressure of water vapor was equal to 5.093 kPa. The gnocchi moisture content trend during storage did not change significantly (*p* > 0.05) across all tested temperatures (4, 8, and 12 °C). The initial moisture content in the samples was 63.7 ± 3.5 g 100 g^−1^ f.w., while at the end of storage (day 67), an average value of 63.4 ± 3.2 g 100 g^−1^ f.w. was recorded. This indicates that 0.3 g of water per 100 g of gnocchi permeated out of the packaging during the storage period, while the temperature had no significant impact on this loss. Therefore, the WVTR value of the packaging is considered acceptable for minimizing water transmission during product storage. The moisture trend contrasts with Lacivita et al. [11], who found a slight decrease in the gnocchi samples during storage. However, the authors did not report the characteristics of the packaging used, and no evaluation can be made about the difference found.

### 3.3. Gas Composition Changes in Headspace of the Packaging

**Figure 1:**
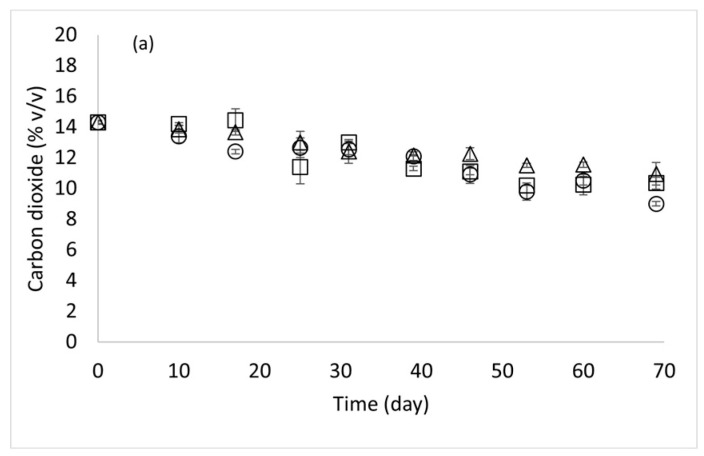

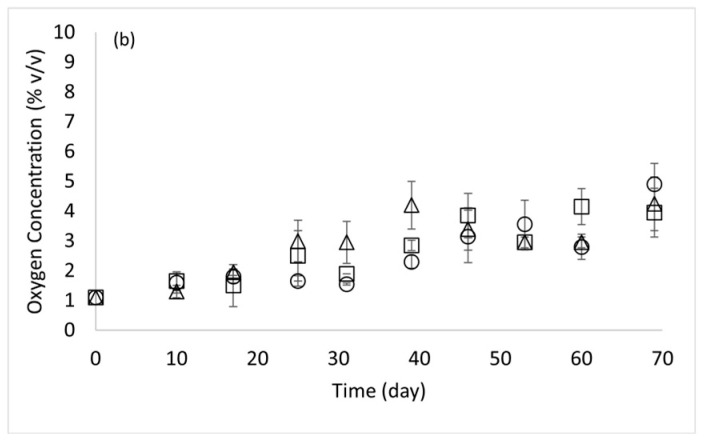
Carbon dioxide (**a**) and oxygen (**b**) changes (% *v v*^−1^) in the headspace of the packaging during gnocchi storage at three different temperatures (circle: 4 °C; triangle: 8 °C; square: 12 °C). [Values are the mean ± standard deviation (*n* = 3)].

Figure 1 illustrates the evolution of O_2_ and CO_2_ concentration (v 100 v^−1^) in the headspace of the packaging, plotted as a function of time at 4, 8 and 12 °C. The oxygen concentration increased over time, reaching approximately 4.5 v 100 v^−1^ at the end of the storage time (Figure 1a), without any statistical difference among the samples stored at different temperatures at the same storage time (*p* > 0.05). O_2_ increase was related to the packaging films’ gas permeability and the MAP equipment’s effectiveness in removing air [36]. On the other hand, the CO_2_ concentration decreased with time, reaching a final value of about 10 v 100 v^−1^ in all samples (Figure 1b). No significant differences were found in relation to storage temperature for the same storage time (*p* > 0.05). This trend depends on gas diffusion through packaging due to the permeability of the plastic material. Notably, while the CO_2_ percentage used for the modified atmosphere in the packaging was 20 v 100 v^−1^, the initial value measured for CO_2_ was 14.3 v 100 v^−1^, and then the gas concentration decreased following a linear trend. This difference may be related to the gas dissolution in the gnocchi during the cooling step of the packed product after the heat treatment [30].

### 3.4. Microbiological Analyses

The initial total viable count of the gnocchi samples was 1.9 log cfu g^−1^. *Listeria* and *Salmonella* were not detected. The microbial load was predominantly represented by aerobic spore-forming bacteria (1.71 log cfu g^−1^), while the anaerobic spore-forming bacteria were present at levels below 1 log cfu. g^−1^. Del Torre et al. [32] found contamination levels lower than 2.0 log cfu g^−1^ even though 33% of the gnocchi samples contained aerobic spore-forming bacteria. Spore-forming bacteria are the microbial group that survive pasteurization treatment and can be found in the product at the end of the processes, potentially causing spoilage [13]. Nevertheless, all microbiological indicators examined did not show growth, and the concentration remained low (average value of approximately 2.5 log cfu g^−1^) throughout the storage periods at all tested temperatures. These results confirm that the pasteurization step and the product’s pH effectively prevented microbial growth throughout the storage period at temperatures below 12 °C [32,33]. Lacivita et al. [11] reported that microbiological quality affects only the secondary shelf life, which occurs when the package is opened, and the gnocchi loses its MAP protection. These results demonstrate that the microbial quality was not critical in the evaluation of the PSL of fresh gnocchi.

### 3.5. Colour Changes

Colour is one of the first attributes perceived by consumers and can strongly influence product acceptability [7]. As expected, raw fresh spinach gnocchi were characterized by high greenness values (a* = −3.30 ± 0.06). Green colour intensity (a*) was chosen to monitor colour changes in fresh spinach gnocchi. No significant changes were found for a* during storage at the different temperatures (Table 2). Regarding the hue angle (*h°*) value, the gnocchi showed an initial value of 178.58 ± 0.01, close to the angle of 180° that represents green hues. No significant differences were observed during storage time at different temperatures (*p* > 0.05).

### 3.6. Cooking Behaviour and Texture Analyses

**Figure 2:**
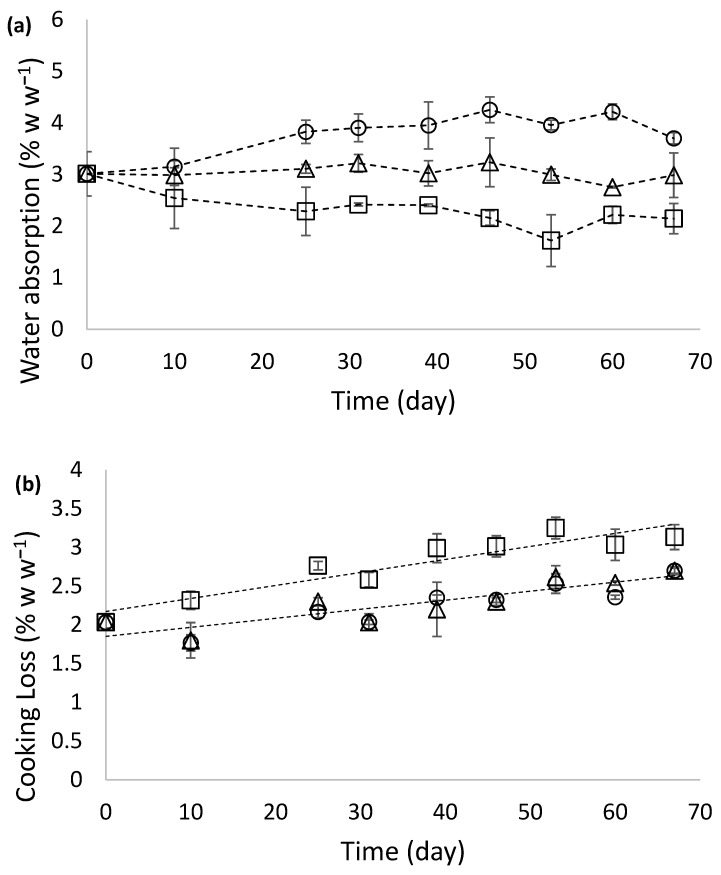
Water absorption index (WAI) in fresh spinach gnocchi stored at different temperatures (4 °C (circle), 8 °C (triangle), and 12 °C (square)) (**a**) and cooking loss from fresh spinach gnocchi stored at different temperatures (4 °C (circle), 8 °C (triangle), and 12 °C (square)) (**b**). [Values are the mean ± standard deviation (*n* = 3)].

The WAI was low for all samples, as shown in Figure 2a. No significant differences over time were found for samples stored at 8 °C. Nevertheless, the gnocchi WAI was statistically different (*p* < 0.05) when stored at 4 °C compared to 12 °C. The WAI ranged from 3.56 ± 0.19 g 100 g^−1^ at 4 °C to 2.74 ± 0.17 g 100 g^−1^ at 12 °C, with no differences observed over time.

The difference in water absorption due to the storage temperature is not surprising considering the starch changes occurring during the preparation of the gnocchi. First, the potato-wheat dough is mixed at 80 °C during the pre-gelatinization step. Then, the dough is shaped into gnocchi and subsequently packed and pasteurized at 70 °C. Due to these steps, the amylose and amylopectin components of starch lose their semicrystalline structure, leading the swelling of the starch granules and the lixiviation of amylose, forming a paste. Upon cooling, the starch polymers tend to recrystallize (retrograde) [37]. According to Chen et al. [38], storage temperature induces differences in the crystalline structure of retrograded potato starch, mainly due to changes in molecular mobility. Moreover, as previously explained by Santagata et al. [39], starch retrogradation can hinder the water absorption process due to the formation of amylose crystals. These hydrophobic crystalline zones are more thermally stable and less prone to absorbing water [40,41]. In the present work, the molecular mobility in samples stored at 4 °C is expected to be slower. Thus, these samples are likely to retrograde with a less efficient crystal formation that would lead to less hydrophobic behaviour and more thermal instability, increasing the water absorption during cooking. Conversely, it would be the opposite for samples stored at 12 °C, where higher molecular mobility could allow more efficient and stable crystal formation.

All the gnocchi samples exhibited low cooking loss values. Significant differences (*p* < 0.05) between the samples stored at 12 °C and those stored at 4 °C and 8 °C were observed. In all cases, cooking loss increased significantly (*p* < 0.05) over time (Figure 2b). This trend persisted throughout the storage period, culminating in cooking loss values of 3.13 ± 0.16 g 100 g^−1^ for samples stored at 12 °C and 2.70 ± 0.05 g 100 g^−1^ and 2.70 ± 0.10 g 100 g^−1^ for samples stored at 4 °C and 8 °C, respectively, by the end of the analysed storage period (Figure 2b). Low transfer of solids into the cooking water is associated with high pasta quality [42]. These results were similar to those reported by Alessandrini et al. [17] (1.72 g 100 g^−1^) and Burgos et al. [4] (1.45 g 100 g^−1^). As previously reported by Bastos et al. [43] for gluten-free spaghetti samples, there is an inverse significant relationship between the WAI and the solid loss during cooking. This relationship was also found in the gnocchi sample stored at 12 °C (*r* = −0.88; *r*^2^ = 0.785; *p* < 0.05). WAI is calculated by weighing samples before and after cooking, while cooking loss is determined by quantifying the mass of solids transferred to the water during cooking. Since water absorption and solid loss occur simultaneously and are both gravimetrically measured, this relationship is expected. High solid losses during cooking reduce the weight of the cooked samples, directly and negatively impacting WAI values. Furthermore, the retrogradation conditions in samples stored at 12 °C, where crystals could be more efficiently formed, could weaken the starch–protein gel formation, contributing not only to a decrease in WAI, as explained above, but also to an increase in solid losses.

**Figure 3:**
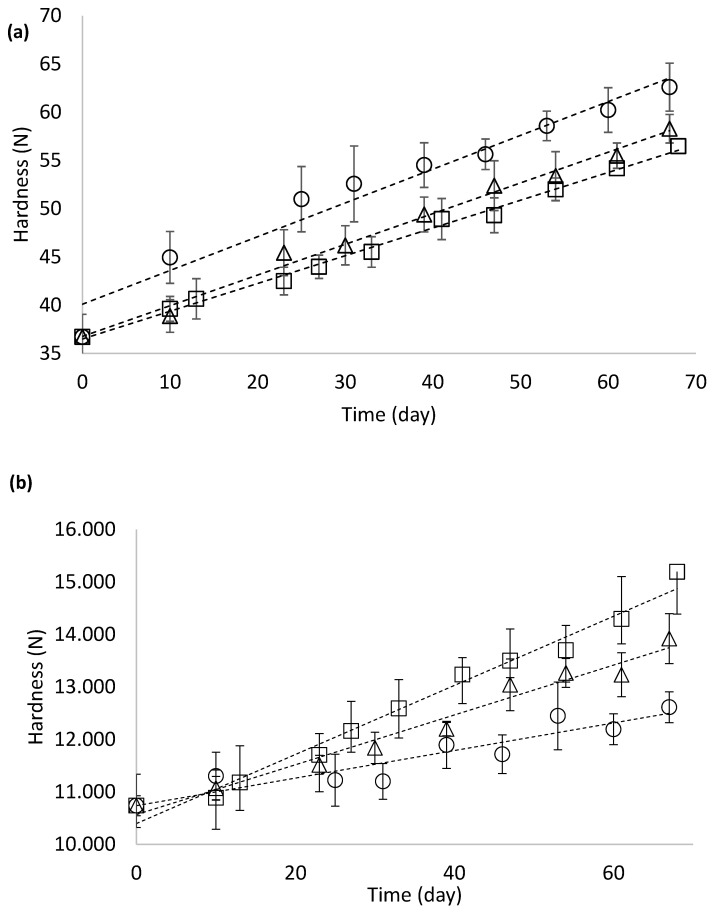
Hardness change in the uncooked (**a**) and cooked spinach gnocchi (**b**), as a function of storage temperature (4 °C (circle), 8 °C (triangle), and 12 °C (square)). [Values are the mean ± standard deviation (*n* = 10)].

The hardness values of the raw gnocchi samples increased during storage at different temperatures (Figure 3a). A strong negative correlation was found between hardness and storage temperature (*p* < 0.05). The results followed a zero-order kinetic trend, and the rate constants (*k*) were calculated (Table 3). The values of *k* (N days^−1^) decreased as the storage temperature increased, suggesting that at low temperature, some matrix modification happened with an increase in the hardness of the product. The amylose present in potato starch can crystallize de novo in refrigerated conditions, with an increase in strength matrix. Amylose retrogradation critically influences the hardness, stickiness and digestibility of a starch gel [44]. This behaviour of amylose during storage is similar to findings by Zardetto et al. [45] regarding fresh pasta, where starch gelatinization occurs at different storage temperatures. The activation energy (*E_a_*) calculated for this process was −3.9 kcal mol^−1^ (*r* = 0.999; *r*^2^ = 0.999; *p* < 0.05). Zardetto et al. [45] reported similar findings for starch in fresh egg pasta, where the *E_a_* was measured at −4.9 kcal mol^−1^.

After cooking, pasta absorbs water, hence developing a softer texture. As shown in Figure 3a,b, the hardness values of all cooked samples were consistently lower than those of the raw samples both at the beginning of storage (10.74 ± 0.38 N and 36.72 ± 4.67 N for the cooked and raw samples on day 0, respectively) and throughout the entire storage period. The hardness reduction between raw and cooked samples agrees with Alessandrini et al. [17]. In addition, in cooked gnocchi, the hardness increased during storage, showing a significant positive correlation with storage temperature (*p* < 0.05) (Figure 3b). Conversely, the hardness decreased in raw fresh gnocchi, reducing the storage temperature.

The WAI and hardness values in raw and cooked samples, all stored at 4 °C, suggest that the partial recrystallization of starch during storage modifies the matrix characteristics. In general, starch retrogradation increases firmness and decreases adhesiveness during storage [44]. Thus, at 4 °C, more rapid crystal formation—albeit with less efficient packing—would be expected, leading to a higher rate of hardness increase in samples stored at this temperature. However, due to the aforementioned inefficient crystal packing, the retrograded starch in these samples would exhibit lower thermal stability and be more easily hydrated during cooking, leading to a softer texture in cooked samples. Conversely, at 12 °C, the crystals formed would be more thermally stable, thereby limiting hydration and further gelatinization during cooking, which would result in a harder texture.

**Figure 4:**
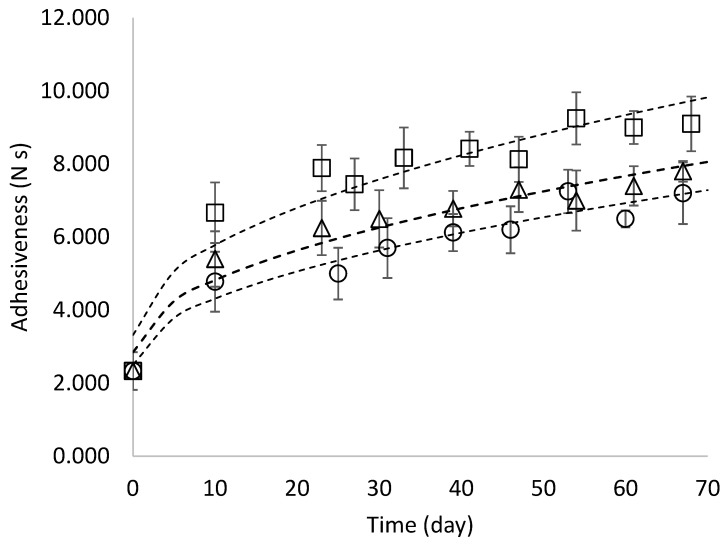
Adhesiveness (N s) of cooked fresh spinach gnocchi as a function of temperature (4 °C (circle), 8 °C (triangle), and 12 °C (square)) during storage)). [Values are the mean ± standard deviation (*n* = 10)].

Adhesiveness measures a product’s stickiness during consumption and is a crucial aspect of pasta texture and quality. When starch granules are released from the pasta into the cooking water, they can gelatinize and coat the product’s surface [46]. In cooked fresh spinach gnocchi, adhesiveness increased during storage time, with a significant effect of storage temperature (*p* < 0.05) (Figure 4). As reported by Khoozani et al. [47], several factors related to starch influence the adhesiveness development in these kinds of products, e.g., quantity, quality, gelatinization state, and matrix in which it is entrapped. Gnocchi samples stored at 4 °C exhibited the lowest adhesiveness after cooking, in agreement with the hardness results. In these samples, water absorption during cooking was higher (Figure 2a), leading to a softer texture (Figure 3b). Under conditions of high hydration, adhesiveness may decrease because excessive water uptake and extensive starch gelatinization can dilute and weaken the surface starch layer, shifting the material response from adhesive to more lubricated behaviour.

### 3.7. Sensory Analysis

**Figure 5:**
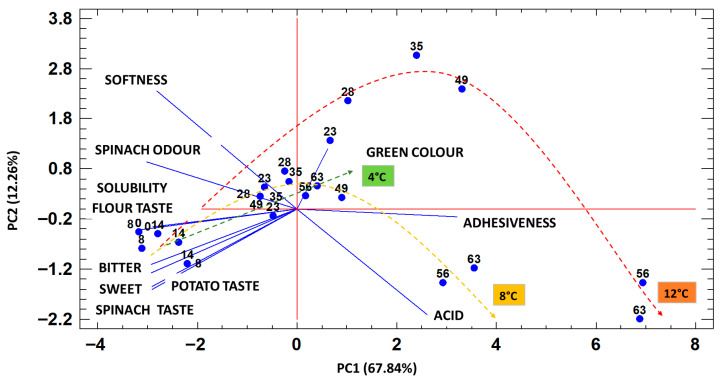
PCA biplot for sensory results of fresh spinach gnocchi stored at three different temperatures (4 °C, 8 °C, and 12 °C). Numbers in symbols refer to storage time (days).

Sensory evaluation of cooked samples was conducted considering the following attributes: colour (green), odour (spinach and potato), taste (spinach, potato, sweet, bitter, and acid), and texture (hardness and adhesiveness). The data were included in a PCA, in which the first two principal components explained 80.1% of the variance (Figure 5). The first component is positively correlated with the adhesiveness parameter and negatively correlated with the sensory attributes of the product’s odour and flavour characteristics (spinach taste and spinach aroma, potato taste). The second principal component is positively correlated mainly with the texture attribute described as softness and negatively with a taste attribute identified as acid. Since the pH value did not increase during the storage time), the sensory perception of acid in the sample stored at 8 °C and, to an even greater degree, at 12 °C is probably due to the reduction in the potato and spinach taste with the formation of an unbalanced sensory profile.

Figure 5 also illustrates the samples’ sensory profile evolution for each storage temperature (4, 8, and 12 °C). As can be seen, the samples stored at 12 °C were characterized by significant changes in sensory attributes during storage after 23 days. Regarding flavour attributes, an increase in the acid attribute and a decrease in the characteristic aromatic notes of the product were observed. Among the texture attributes, softness decreased, while adhesiveness increased, becoming extremely intense. The sensory profile of the product stored at 8 °C showed significant variations toward the end of the storage period, particularly after 49 days, with adhesiveness also becoming remarkably intense, especially in the last stage of the storage period. The aromatic notes of spinach and potato decreased during storage and were barely perceptible at the final evaluation for both the 8 and 12 °C stored samples. Among the basic tastes, there was an increase in acidity and bitterness during the storage period.

**Figure 6:**
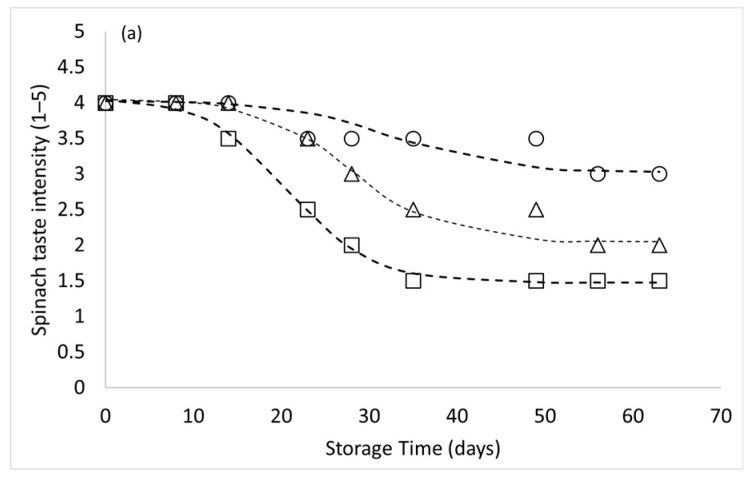

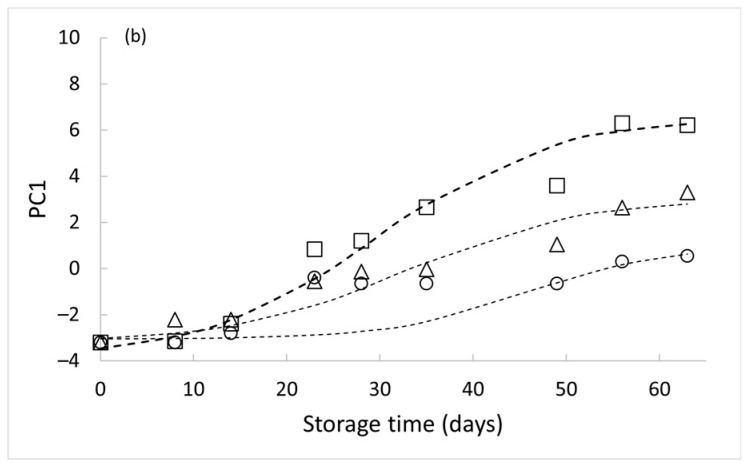
Spinach taste score kinetics during storage at three different temperatures (4 °C (circle), 8 °C (triangle), and 12 °C (square)) (**a**) and variation of the PC1 as a function of the storage time at the different storage temperatures (4 °C (circle), 8 °C (triangle), and 12 °C (square)) (**b**).

The sensory profile of samples stored at 4 °C showed less pronounced variation along the principal components. The product underwent a slight decrease in characteristic aromatic notes (spinach and potato notes). The acidic taste, strongly affected in the samples stored at 8 and 12 °C, increased only slightly, maintaining a medium–low intensity even at the end of the storage period. Slight changes were observed in these samples for the softness parameter. Similarly, regarding adhesiveness, which is highly discriminating for samples stored at 8 and 12 °C, the variation was slight even at the end of the storage period. Two attributes with opposite effects, the spinach taste (negative coordinate on PC1) and adhesiveness (positive coordinate on PC1), contributed to the discrimination of the samples by sensory analysis during storage time. The trend of panel score vs. storage time was considered to define the stability time for spinach taste maintenance. Figure 6a shows these data modelled by Equation (4).

The results confirmed that sensory indices played the dominant role in differentiating the stages of product degradation. Therefore, PC1 (samples scores) was analysed as a function of storage time (Figure 6b). The second derivative of this relation was used to determine the maximum acceptable time based on sensory results, which can be considered the “stability” time [48]. To determine PSL, the OQI (according to Equation (3)) was also used. A value of 6.5 on the 10-point scale was set as the acceptability limit for PSL estimation. This threshold was derived from values reported in the literature [16]. Bianchi et al. [27] considered an Overall Quality Index of 6 on a 9-point scale as the acceptability limit, which corresponds to 6.5 on the 10-point scale used in this study. The panel confirmed the appropriateness of this choice. Although the sensory panel evaluated multiple quality attributes, panellists were also asked at each session to classify each sample as acceptable or unacceptable. The results were then compared with the Overall Quality Index, and all samples judged “not acceptable” had an OQI < 6.5. Moreover, a significant correlation was found between the principal component primarily discriminating degraded samples (PC1) and OQI (*r* = −0.993; *r*^2^ = 0.985; *p* < 0.05). These results corroborate the methodology used to calculate OQI and support its application in PSL estimation.

Figure 6b presents the PC1 scores for each storage temperature alongside the predicted curves generated with the sigmoidal model described in Equation (4). PSL calculated by the minimum of the second derivative of Equation (4) was 56 ± 3 days at 4 °C, 45 ± 1 at 8 °C and 38 ± 2 at 12 °C. From the sensory analysis results, the estimated PSL model at all storage temperatures (4, 8 and 12 °C) was calculated. A high correlation was obtained in the regression of experimental value for OQI (*r* = −0.99; *r*^2^ = 0.983; *p* < 0.05) and PC1 score analysis (*r* = −0.99; *r*^2^ = 0.994; *p* < 0.05). In addition, the values of *E_a_* and *Q*_10_ were calculated by Equation (5) and Equation (7), respectively.

**Figure 7:**
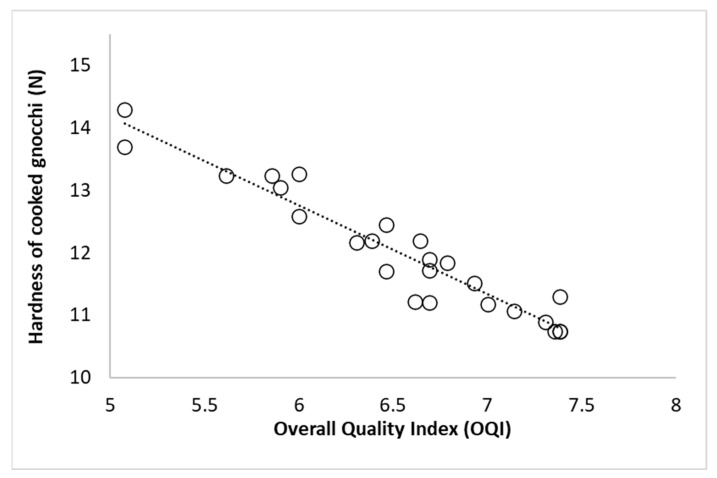
Relationship between Overall Quality Index (OQI) and hardness parameter (N) in cooked fresh spinach gnocchi.

Gnocchi quality is usually judged by its texture [7,17]. Cooked gnocchi with a soft, non-adhesive texture are considered to be similar to homemade products because they are made with steam-cooked potatoes. Texture was one of the major contributors to the linking and rejection of the sample. The PCA biplot showed that softness and adhesiveness were the main attributes driving sample discrimination along the principal component (Figure 5). For this reason, the hardness value of cooked gnocchi was correlated with the OQI (*r* = −0.95; *r*^2^ = 0.912; *p* < 0.05) (Figure 7). Most samples exhibited hardness values between 10.5 and 13.0 N, and, using the OQI acceptability limit of 6.5, the corresponding hardness rejection value was calculated as 12.1 N.

Hence, the hardness of cooked gnocchi was identified as the critical parameter for developing the PSL model at all storage temperatures (4, 8, and 12 °C). The PSL was calculated using the parameters from the experimental results, which followed zero-order kinetics at the three temperatures, as illustrated in Figure 3b. The *Q*_10_ value calculated by Equation (7) was 3.0; from this value, the activation energy (*E_a_*) calculated using Equation (8) was 75 kJ mol^−1^. These values agree with Olivera and Salvadori [49], who reported a *Q*_10_ value equal to 2.52 and *E_a_* 51 kJ mol^−1^ for the evaluation of consistency in lasagna samples.

Table 4 summarizes the maximum stability time (*t_s_*) estimated at each temperature for the critical indicators identified (hardness and sensory analysis), the method used for their calculation, and the values of *E_a_* and *Q*_10_ obtained. It is evident from the results that among the different storage temperatures, some criteria were stricter than others.

In addition, comparing the *Q*_10_ values obtained revealed that texture is more sensitive to temperature changes. Moreover, from a PSL point of view, the hardness criterion could be used to determine the PSL of the fresh vegan spinach gnocchi at given time–temperature combinations. Considering the foreseeable storage, distribution, and use conditions reported by Zardetto [50] for refrigerated products, the PSL of vegan fresh spinach gnocchi was estimated using the time–temperature tolerance (TTT) approach. The shelf-life fraction consumed at each stage was calculated by the shelf-life plot obtained using the hardness criterion, and the PSL determined was 42 ± 3 days.

## 4. Conclusions

The primary shelf life (PSL) of fresh vegan spinach gnocchi packaged under a modified atmosphere (MAP) was evaluated at three different storage temperatures. The results showed that, under the applied MAP conditions (20% CO_2_ and O_2_ < 1.5%), sensory and texture attributes were the key factors in determining PSL as a function of storage temperature. In contrast, microbiological criteria were not suitable for assessing the PSL of refrigerated fresh potato gnocchi, as they were characterised by a pH < 5.0.

Storage-related changes in hardness followed zero-order kinetics, and the Arrhenius equation adequately described the temperature dependence of the kinetic constants. This approach enabled the estimation of relevant physicochemical parameters, including Q_10_ and activation energy (Eₐ).

Hardness of the cooked product proved to be a reliable indicator of deterioration, showing a strong correlation with the Overall Quality Index (OQI) determined by the sensory panel. The agreement among the different shelf-life estimation methods was influenced by storage temperature; however, across all tested temperatures, hardness-based indices and sensory approaches (PCA and OQI) exhibited good consistency.

When the OQI threshold was set at 6.5, overall acceptability values confirmed that this parameter was appropriate as a shelf-life indicator. The close agreement between hardness-based indices and sensory OQI further supports the use of instrumental texture measurements as an in-process quality control tool to ensure sensory-perceived quality.

The significant temperature dependence of hardness and sensory kinetics underscores the importance of storing the product at or below 4 °C, with minimal temperature fluctuations (±2 °C). Deviations above this threshold markedly accelerate quality degradation, significantly reducing the PSL. These results highlight the importance of the storage conditions reported on the labelling, which should emphasise strict refrigerated storage requirements. At the same time, distribution logistics should prioritise maintaining an uninterrupted cold chain to ensure the declared shelf life is met under commercial conditions.

Finally, when considering the hardness evolution of the cooked product under realistic storage, distribution, and consumer-use conditions for refrigerated foods, the time–temperature tolerance (TTT) approach allowed estimation of a PSL of 42 ± 3 days.

Further studies are needed to verify whether the sensory panel results align with consumer acceptance tests and whether the identified threshold corresponds to actual consumer unacceptability.

## Figures and Tables

**Table 1:** Proximate composition of raw fresh spinach gnocchi.

	Composition (g 100 g^−1^, Fresh Weight)
Protein	3.3 ± 0.3
Fiber	2.0 ± 0.1
Fat	0.4 ± 0.1
Carbohydrate	29.4 ± 0.2
Moisture	63.7 ± 0.8
Ash	1.2 ± 0.3

Values are the mean ± standard deviation (*n* = 3).

**Table 2:** Colour parameters of the raw fresh spinach gnocchi as a function of time and storage temperature.

Time (Days)	Storage Temperature (°C)					
	4	8	12	4	8	12
	a*			*h°*		
0	−3.30 ± 0.06	−3.30 ± 0.06	−3.30 ± 0.06	178.58 ± 0.01	178.58 ± 0.01	178.58 ± 0.01
10	−3.59 ± 0.02	−3.61 ± 0.02	−3.61 ± 0.01	178.60 ± 0.02	178.60 ± 0.01	178.62 ± 0.01
34	−3.76 ± 0.01	−3.55 ± 0.01	−3.79 ± 0.19	178.61 ± 0.01	178.60 ± 0.02	178.61 ± 0.02
54	−3.66 ± 0.06	−3.86 ± 0.08	−3.86 ± 0.08	178.60 ± 0.01	178.60 ± 0.01	178.60 ± 0.01
67	−3.66 ± 0.04	−3.70 ± 0.08	−3.70 ± 0.06	178.61 ± 0.01	178.60 ± 0.02	178.62 ± 0.04

Values are the mean ± standard deviation (*n* = 3).

**Table 3:** Kinetic parameters for hardness in fresh spinach gnocchi as a function of storage temperature.

Temperature (°C)	*k_Har_* (N Days^−1^)	Intercept	*r*	*r* ^2^	*p*
4	0.35 ± 0.03	40.10	0.98	0.955	<0.01
8	0.32 ± 0.01	36.76	0.99	0.993	<0.01
12	0.29 ± 0.01	36.49	0.99	0.995	<0.01

*k_Har_*: hardness kinetic constant rate. Values are the mean ± standard deviation.

**Table 4:** Estimated stability time (*t_s_*), *E_a_* and *Q*_10_ values for hardness and sensory analysis of fresh vegan spinach gnocchi stored at 4, 8, and 12 °C.

Analysis	Definition of *t_s_*	Estimated *t_s_*			*E_a_* (kJ mol^−1^)	*Q*_10_
		4 °C	8 °C	12 °C		
Sensory analysis	*d^2^*(*PC1*)/*dt*^2^	56 ± 3	45 ± 1	38 ± 2	32	1.62
Sensory analysis	OQI	58 ± 2	43 ± 3	27 ± 2	63	2.60
Hardness	Limit 12.1 N	55 ± 2	35 ± 2	27 ± 2	75	3.00

*d*^2^(*PC1*)/*dt*^2^ = second derivative of the PC1 vs. *t* plot. OQI = Overall Quality Index.

## Data Availability

The original contributions presented in this study are included in the article. Further inquiries can be directed to the corresponding author.
